# CAR designs for solid tumors: overcoming hurdles and paving the way for effective immunotherapy

**DOI:** 10.52601/bpr.2023.230020

**Published:** 2023-10-31

**Authors:** Yuanbin Cui, Mintao Luo, Chuanyuan Gu, Yuxian He, Yao Yao, Peng Li

**Affiliations:** 1 China-New Zealand Joint Laboratory of Biomedicine and Health, State Key Laboratory of Respiratory Disease, CAS Key Laboratory of Regenerative Biology, Guangdong Provincial Key Laboratory of Stem Cell and Regenerative Medicine, Guangzhou Institutes of Biomedicine and Health, Chinese Academy of Sciences, Guangzhou 510530, China; 2 University of California San Diego, La Jolla, CA 92093-0021, USA

**Keywords:** Chimeric antigen receptor T cell, Solid tumor, Immunotherapy

## Abstract

Chimeric antigen receptor T cell (CAR-T) therapy has revolutionized immunotherapy by modifying patients' immune cells genetically. By expressing CARs, these modified cells can specifically identify and eliminate tumor cells. The success of CAR-T therapy in hematological malignancies, such as leukemia and lymphoma, has been remarkable. Numerous studies have reported improved patient outcomes and increased survival rates. However, the application of CAR-T therapy in treating solid tumors faces significant challenges. Solid tumors possess complex microenvironments containing stromal cells, extracellular matrix components, and blood vessels. These factors can impede the infiltration and persistence of CAR-T cells within the tumor. Additionally, the lack of target antigens exclusively expressed on tumor cells raises concerns about off-target effects and potential toxicity. This review aims to discuss advancements achieved by CAR-T therapy in solid tumors and the clinical outcomes in the realm of solid tumors.

## INTRODUCTION

The structure of a chimeric antigen receptor (CAR) consists of three main components: an extracellular targeting domain, a transmembrane domain, and an intracellular signaling domain. The extracellular targeting domain is responsible for recognizing specific antigens on the surface of cancer cells. It is usually derived from an antibody or antibody fragment, which is selected to bind specifically to a particular cancer-associated antigen. This targeting domain allows CAR-T cells to recognize and engage cancer cells with high specificity. The transmembrane domain is a short segment of amino acids that crosses the cell membrane. It anchors the CAR protein within the outer membrane of the cell, enabling it to remain attached to the cell surface. This domain provides stability and structural integrity to the entire CAR construct. The intracellular signaling domain is responsible for activating immune cells upon engagement with cancer cells. It contains components that initiate a signal cascade, leading to the destruction of the cancer cell. T-cell activation requires three essential signals. Firstly, T-cells recognize specific antigens presented by antigen-presenting cells (APCs) through their T-cell receptors (TCRs). This antigen recognition is crucial for initiating T-cell activation. Secondly, costimulatory signals are necessary to provide a second signal for complete T-cell activation. Molecules like CD80 and CD86 on APCs bind to CD28 on T-cells, delivering the needed co-stimulation. Lastly, the third signal for T-cell activation comes from pro-inflammatory cytokines or signals from surrounding cells, phagocytes, or other immune cells such as dendritic cells. Common pro-inflammatory cytokines like IL-1, IL-6, and TNF-α have an additional reinforcing effect. They aid in activating T-cells, promoting their proliferation, and facilitating their differentiation into effector cells. The most common signaling domains used in CARs are derived from TCR signaling molecules, such as CD3ζ, combined with one or more costimulatory domains, such as CD28 or 4-1BB. These domains enhance the activation and persistence of CAR-T cells.

**Figure 1:**
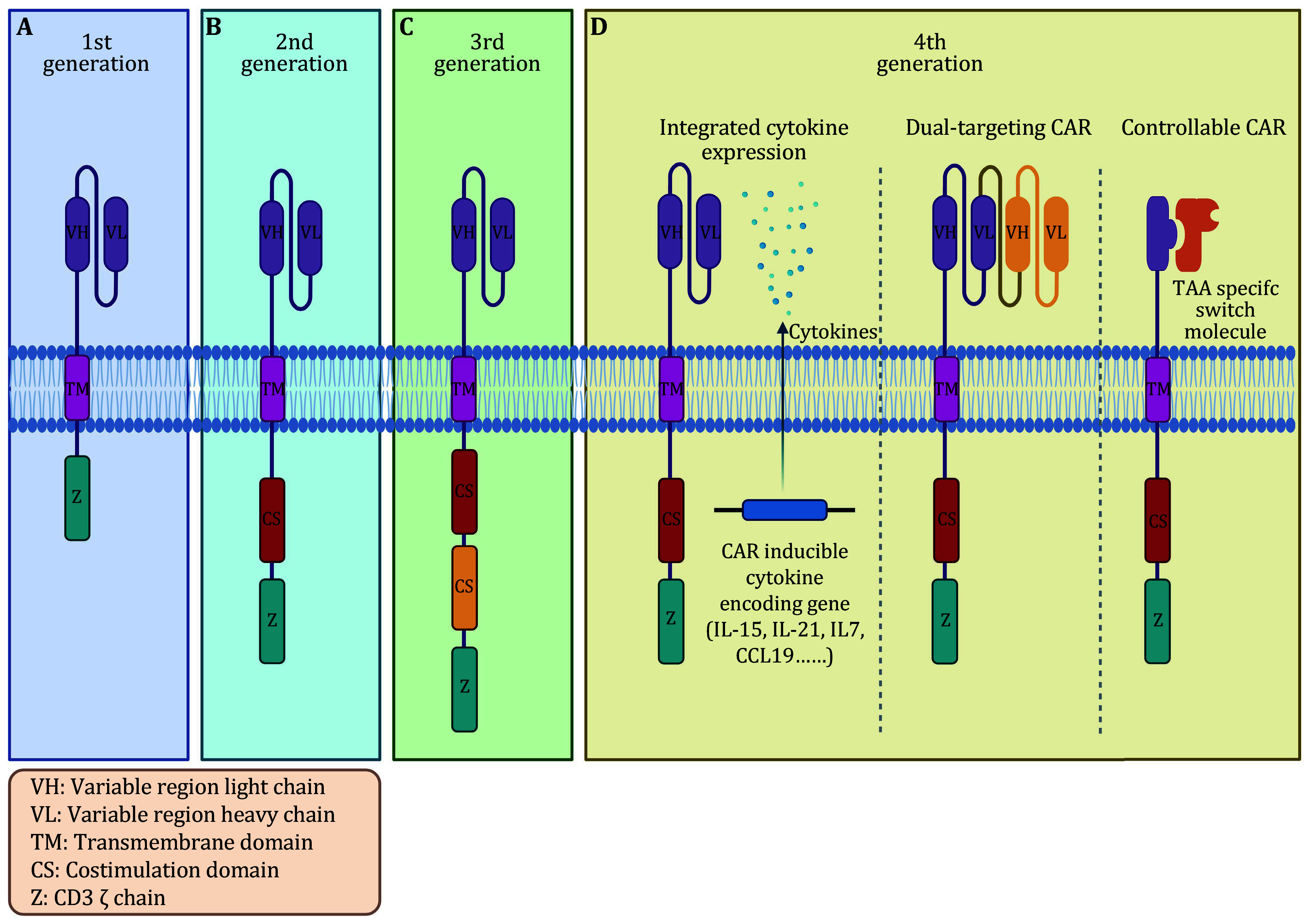
Schematic diagram of CAR design development. The figure illustrates the progression of CAR design through different generations. A CAR structure consists of three main components: an extracellular targeting domain, a transmembrane domain, and an intracellular signaling domain. The targeting domain is usually derived from the variable region of the antibody. The transmembrane domain anchors the CAR protein within the cell membrane. The signaling domain initiates a signal cascade for cancer-cell destruction. **A** First-generation CAR-T cells. Initially developed with a single signaling domain (derived from the CD3ζ chain), these CARs successfully redirected T cells to target cancer cells. However, their clinical efficacy was limited due to insufficient expansion and persistence in patients, especially against solid tumors. **B** Second-generation CAR-T cells. To enhance functionality and persistence, second-generation CAR-T cells were engineered by incorporating an additional costimulatory domain (*e*.*g*., CD28 or 4-1BB) along with the CD3ζ signaling domain. This modification significantly improved CAR-T expansion, cytokine production, proliferation, and antitumor activity. **C** Third-generation CAR-T cells. To further enhance potency, third-generation CAR-T cells incorporated multiple costimulatory domains alongside the CD3ζ signaling domain. These CAR-T cells aimed to overcome tumor evasion mechanisms and improve persistence. **D** Fourth-generation CAR-T.Building upon previous generations, fourth-generation CAR-T-cell therapy seeks to enhance effectiveness and safety through additional functionalities or modifications. Several key features have been proposed. Integrated cytokine expression: some fourth-generation CAR-T designs include genetic modifications enabling the secretion of specific cytokines, such as IL-21, IL-15, IL-7, and CCL19. The secretion of such cytokines can enhance the antitumor immune response by recruiting and activating other immune cells. Dual-targeting CAR-T cells: fourth-generation CAR-T cells can be engineered to simultaneously recognize multiple tumor-associated antigens. This approach aims to overcome antigen escape and reduce the likelihood of cancer relapse by targeting distinct epitopes on tumor cells. Controllable CAR-T cells: strategies have been explored to enable conditional activation or suppression of CAR-T cells, providing better control over their activity. Inducible switches can regulate CAR expression or function in response to external stimuli such as small molecules or antibodies

Four generations of CAR-T cells have been developed to date (Fig. 1). The first generation of CAR-T cells consisted of a single signaling domain, typically derived from the CD3ζ chain of the T-cell receptor complex. These cells were successful in redirecting T cells to recognize cancer cells, but their efficacy was limited due to inadequate expansion and persistence after infusion into patients. First-generation CAR-T cells demonstrated modest clinical responses but had significant challenges in terms of durability and effectiveness against solid tumors. To enhance the functionality and persistence of CAR-T cells, second-generation CAR-T cells were engineered with an additional costimulatory domain along with the CD3ζ signaling domain. Costimulatory domains such as CD28 or 4-1BB were incorporated to provide additional activation signals to T cells, thus promoting proliferation and cytokine production and improving survival. This modification significantly increased CAR-T expansion, persistence, and antitumor activity. Second-generation CAR-T cells have notably advanced the field and shown tremendous clinical success, particularly in the treatment of B-cell malignancies. The third generation of CAR-T cells further enhanced their potency by incorporating multiple costimulatory domains, aiming to overcome tumor evasion mechanisms and improve persistence. These CAR-T cells typically contain two different costimulatory domains along with the CD3ζ signaling domain. Third-generation CAR-T cells have demonstrated improved expansion and persistence in preclinical models, but their clinical benefits have yet to be fully realized compared to second-generation CAR-T cells. Research is ongoing in this area. Fourth-generation CAR-T-cell therapy builds upon the foundation of previous generations by incorporating additional functionalities or modifications to further enhance the effectiveness and safety of this immunotherapy. While there is not a universally defined set of characteristics for fourth-generation CAR-T cells, several key features have been proposed.

(1) Integrated cytokine expression. Some fourth-generation CAR-T-cell designs include genetic modifications that allow them to secrete specific cytokines, such as interleukin-21 (IL-21), interleukin-15 (IL-15), interleukin-7 (IL-7), and C-C motif chemokine 19 (CCL19). The secretion of IL-15 can potentially enhance the antitumor immune response by recruiting and activating other immune cells (Adachi *et al.* 2018; Batra *et al.* 2020; Duan *et al.* 2021; Lanitis *et al.* 2021; Pang *et al.* 2021).

(2) Dual-targeting CAR-T cells. Fourth-generation CAR-T cells can be engineered to recognize multiple tumor-associated antigens simultaneously. This approach aims to overcome antigen escape and reduce the chances of cancer relapse by targeting different epitopes on tumor cells.

(3) Controllable CAR-T cells. Strategies have been explored to enable conditional activation or suppression of CAR-T cells to provide better control over their activity. These approaches involve adding inducible switches that can regulate CAR expression or function in response to external stimuli such as small molecules or antibodies.

As research progresses, further advancements in CAR-T cells are likely to occur, leading to more refined and effective treatments for various types of cancers.

## EXTRACELLULAR DESIGN OF CARS FOR TARGETING SOLID TUMORS

### Extracellular domain to recognize solid tumor antigens

CAR-T therapy has emerged as a promising treatment modality for solid tumors, offering a targeted approach to combat various types of cancer. The extracellular domain of CAR plays a crucial role in recognizing specific antigens present in solid tumors. By targeting these antigens, CAR-T cells can be directed toward solid tumors, offering a targeted approach to cancer treatment.

Selecting a suitable tumor antigen is crucial in CAR design. CAR targeting antigen should be highly expressed in cancer cells while being minimally expressed, or absent, in healthy tissues to enable specific targeting and reduce off-target toxicity. Several specific antigens have been identified as potential targets for CAR-T therapy in solid tumors. One such antigen is CD133, which has been found in glioblastoma, hepatocellular carcinoma, and lung cancer. Studies (Bueno *et al.* 2019; Dai *et al.* 2020; Vora *et al.* 2020) have shown the potential of CAR-T cells targeting CD133 in improving outcomes for these challenging malignancies. Another promising antigen is MSLN, which is expressed in various types of cancer. CAR-T cells targeting MSLN have shown encouraging results in treating mesothelioma (Adusumilli *et al.* 2021; Castelletti *et al.* 2021), lung cancer (Adusumilli *et al.* 2021), breast cancer (Adusumilli *et al.* 2021; Schoutrop *et al.* 2021), gastric cancer (Lv *et al.* 2019; Zhang *et al.* 2019; Zhao *et al.* 2021), and pancreatic cancer (Beatty *et al.* 2018; Good *et al.* 2021; Pang *et al.* 2021), highlighting the versatility and potential of MSLN-targeted CAR-T therapy in multiple solid tumors. EGFR1 and EGFR2 (HER2) are also promising targets, particularly in breast cancer (Xia *et al.* 2021; Zhou *et al.* 2022) and glioblastoma (Choi *et al.* 2019). Preclinical and clinical studies have shown promising results for CAR-T therapy directed against EGFR1, offering a potential breakthrough in treating these challenging cancers. EGFR2-targeted CAR-T therapy has shown promising outcomes in melanoma (Forsberg *et al.* 2019) and medulloblastoma (Vitanza *et al.* 2021), providing hope for its application in the treatment of solid tumors. In gastrointestinal cancer, Claudin18.2 represents an attractive target for CAR-T therapy. Claudin18.2 (CLDN18.2)-redirected CAR-T cells showed promising efficacy against gastric cancer (GC) in various preclinical studies (Cao *et al.* 2022; Jiang *et al.* 2019; Qi *et al.* 2022). Alpha-fetoprotein (AFP) has been reported as a potential target for liver cancer (Hu *et al.* 2022; Liu *et al.* 2017; Wang *et al.* 2023). A study demonstrated that CAR-T AFP can elicit a potent antitumor response in liver cancer. MAGE-A1 has proven effective against lung adenocarcinoma (Mao *et al.* 2019), and PSMA has shown promising results in prostate cancer (Kloss *et al.* 2018; Narayan *et al.* 2022). TEM-8 has shown promise as a target in triple-negative breast cancer (Byrd *et al.* 2018) and gastric adenocarcinoma (Sotoudeh *et al.* 2019). GD2-targeted CAR-T cells have demonstrated efficacy in treating neuroblastoma (Caforio *et al.* 2021; Del Bufalo *et al.* 2023; Moghimi *et al.* 2021), diffuse midline gliomas (Majzner *et al.* 2022; Moghimi *et al.* 2021; Mount *et al.* 2018), and glioblastoma (Gargett *et al.* 2022), offering new possibilities for the treatment of these challenging solid tumors.

Most of the current CAR-T designs have focused on targeting surface antigens expressed on normal cells, which can lead to the "on target off tumor" effect, which is a significant concern in the field. Moreover, the presence of abundant antigens *in vivo* can result in overstimulation of CAR-T cells and contribute to severe immune toxicity (Song and Milone 2021). However, tumors associated with viral infections offer an opportunity to explore viral antigens that are not expressed on the surface of normal cells for CAR-T engineering. One such viral antigen is the gp350 envelope protein, which exhibits high expression on infected cells during Epstein–Barr virus (EBV) lytic reactivation and sporadic expression on the surface of latently infected cells. This makes it a potential therapeutic target for designing CAR-T therapy specifically aimed at combating the virus. Recent findings support the innovative use of gp350-specific CAR-T cells in treating EBV-associated solid tumors such as nasopharyngeal carcinoma (Zhang *et al.* 2023). Additionally, CAR-T cells targeting the hepatitis B virus (HBV) surface protein have demonstrated promising antitumor effects in hepatocellular carcinoma (HCC) organoids (Zou *et al.* 2021). These studies highlight the substantial potential of utilizing virus-specific antigens present in solid tumors as valuable targets for advancing CAR-T therapy and opening up new avenues for effective treatment strategies.

In summary, the extracellular domain of CAR plays a crucial role in recognizing specific antigens in solid tumor cells. The availability of diverse targets, such as CD133, MSLN, EGFR, HER2, Claudin18.2, AFP, MAGE-A1, PSMA, TEM-8, GD2 and virus-related antigens, highlights the tremendous potential of CAR-T therapy in various types of solid tumors. Tumor antigens are specific molecules or proteins expressed on the surface of tumor cells and serve as targets for CAR-T therapy. These antigens often exhibit characteristics such as overexpression on cancer cells compared to normal cells, tumor-specificity in their expression, and playing essential roles in tumor growth, survival, or metastasis. Developing novel tumor antigens for CAR-T therapy should involve comprehensive characterization, assessing antigenicity, specificity, and expression levels across different tumor subtypes. Safety considerations are crucial to avoid unintended targeting of normal tissues, while efficacy assessment through preclinical models and clinical trials helps evaluate the ability of CAR-T cells targeting these antigens to effectively eliminate tumors and induce long-term remission. By adhering to these principles, additional studies and clinical trials can develop targeted and effective novel tumor antigens for CAR-T therapy.

### CAR design strategies to overcome antigen heterogeneity in solid tumors

Antigen heterogeneity in solid tumors refers to the variation in the expression of antigens on the surface of tumor cells. This heterogeneity can lead to immune evasion, as tumor cells with different antigen profiles may escape immune recognition. It also poses challenges for immunotherapies that target specific antigens, as some tumor subpopulations with low or absent antigen expression may remain unaffected. Clonal evolution can contribute to antigen heterogeneity by introducing genetic changes during tumor progression. Addressing antigen heterogeneity is a critical aspect of developing effective therapies against solid tumors. The fundamental principle of overcoming tumor antigen heterogeneity in solid tumors for CAR-T therapy is to design CAR that can effectively target multiple antigens simultaneously. This strategy increases the likelihood of recognizing and killing cancer cells with varying antigen profiles, addressing the immune evasion caused by antigen heterogeneity.

One approach to overcome antigen heterogeneity is to design CAR-T cells that target multiple antigens simultaneously. This strategy increases the likelihood of tumor cell recognition and killing. A study demonstrated the efficacy of dual-targeting CAR-T cells against both HER2 and IL13Rα2 antigens in glioblastoma models, resulting in enhanced antitumor activity (Hegde *et al.* 2016). Recent studies have demonstrated the efficacy of bispecific CAR-T cells targeting both CD19 and CD20 antigens in lymphoma, resulting in improved tumor control (Larson *et al.* 2023; Shah *et al.* 2020; Tong *et al.* 2020).

CD70 and B7-H3 are proteins involved in immune regulation that are present on the surface of various cancer cells. These proteins serve as potential targets for cancer immunotherapy, where blocking or targeting them with CAR-T therapy can restore immune recognition and enhance the antitumor response. A study investigated the efficacy of bivalent tandem CAR-T (TanCAR) cells against tumors both *in vitro* and *in vivo*. The findings revealed coexpression of CD70 and B7-H3 in multiple tumor types, with the CD70-targeted CAR exhibiting a stronger affinity and superior antitumor effect. TanCAR-T cells displayed enhanced cytolytic ability and cytokine release when encountering tumor cells expressing both CD70 and B7-H3. Even at low doses, TanCAR-T cells effectively controlled lung cancer and melanoma xenografts, leading to improved overall survival rates. These findings highlight the enhanced antitumor functionality of TanCAR-T cells targeting CD70 and B7-H3, addressing the challenges associated with antigenic heterogeneity and variation in solid tumor treatment (Yang *et al.* 2020a).

NKG2D is a natural killer (NK) cell receptor that recognizes stress-induced ligands expressed in cancer cells. The engagement of NKG2D with its ligand triggers NK cell-mediated cytotoxicity against tumor cells. However, cancer cells can evade this immune response by downregulating NKG2D ligands or shedding them from the cell surface. To overcome this, researchers have explored the use of DAP10, an adaptor protein that enhances NKG2D signaling. DAP10 overexpression in CAR-T cells has been shown to enhance their cytotoxicity against NKG2D ligand-deficient tumor cells (Niu *et al.* 2019). This approach can potentially address the heterogeneity of NKG2D ligand expression in solid tumors and improve CAR-T therapy outcomes.

### CAR design strategies to enhance control over CAR-T activity in solid tumors

Switchable CAR-T therapies offer a promising avenue to enhance control over CAR-T activity, combat cancer cell heterogeneity, and alleviate off-target effects associated with conventional CAR-T treatments. By incorporating switches into CAR-T designs, the responses of CAR-T cells can be controlled through dosage modulation.

These novel CAR-T designs incorporate an inducible safety switch, allowing for controlled modulation of their activity. By activating the safety switch, CAR-T cells can be temporarily deactivated in response to adverse events or adjust their activity according to the tumor microenvironment. This dynamic approach offers flexibility in targeting diverse tumor cell populations and managing potential toxicities (McCue *et al.* 2022).

Encouraging results have been observed in HER2-expressing breast cancers using switch molecules employing FITC or peptide neo-epitopes. These switch molecules successfully redirect CAR-T cells toward HER2-expressing tumor cells, resulting in complete tumor clearance in preclinical models (Cao *et al.* 2016).

Another exemplary design is the versatile adapter CAR-T (AdCAR-T). Adapter molecules specific to cancer can redirect AdCAR-T cells toward alternative target antigens beyond the primary antigen CD19. In cases where CD19-negative lymphoma subsets are present, AdCAR-T cells remain functional by targeting antigens such as CD20, CD22, CD79B, and ROR-1. The ability to modulate CAR specificity via the exchange of adapter molecules expands the application of CAR-T therapy, significantly enhancing its effectiveness against leukemia and lymphoma (Atar *et al.* 2022).

The development of biotin-binding immune receptors (BBIRs) has revolutionized the recognition specificity capabilities of bioengineered lymphocyte populations. BBIR T cells exclusively recognize and bind to cancer cells pre-targeted with specific biotinylated molecules, enabling simultaneous or sequential targeting of multiple distinct antigens. By incorporating multiplex antigen expression profiles, this universal T-cell specificity platform extends conventional CAR-T approaches, resulting in tailored T cells with unlimited antigen specificity for improved efficacy in adoptive T-cell immunotherapies against cancer (Urbanska *et al.* 2012).

Another elegant strategy involves the utilization of anti-tag CARs (AT-CARs), which bind to tags present on tumor-targeting antibodies. Monomeric streptavidin 2 (mSA2) biotin-binding domain powers T cells expressing mSA2 CARs, enabling them to effectively target cancer cells coated with biotinylated antibodies specific to certain tumor-associated antigens. This universal AT-CAR-T approach can be combined with various biotinylated tumor-specific antibodies, potentially expanding its applicability across diverse tumor types (Lohmueller *et al.* 2017).

UniCAR-T technology represents another modular CAR platform that broadens recognition specificity beyond tumor-associated antigens (TAAs). Instead of directly targeting TAAs, UniCAR-T cells recognize unique peptide epitopes on engineered recombinant targeting modules (TMs). This approach facilitates efficient retargeting of UniCAR-T cells to different tumors by utilizing TMs specific to those tumors. Notably, nanobody-based TMs against EGFR-positive tumors have demonstrated highly effective and target-dependent tumor cell lysis. The dissociation of UniCAR-T TM complexes allows repeated stop-and-go retargeting of tumor cells, providing versatility via UniCAR-T technology (Albert *et al.* 2017).

Additionally, the split, universal, and programmable (SUPRA) CAR system combines multiple advanced features into a single integrated system. This system enables target switching without re-engineering T cells, fine-tuning T-cell activation strength, and detection and response to multiple antigens. Demonstrating broad utility across diverse tumor models, the humanized components of the SUPRA CAR system minimize potential immunogenicity concerns. Furthermore, it can independently regulate distinct subsets of T cells, highlighting a dually inducible CAR system (Cho *et al.* 2018).

Furthermore, the expression of chimeric Fc receptors (CFRs), composed of extracellular regions of Fc receptors, the 4-1BB endodomain, and the CD3z domain, enables T cells with antibody-dependent cell-mediated cytotoxicity (ADCC) capabilities to eradicate lymphoma and breast cancer cells in the presence of rituximab and trastuzumab, respectively (Cui *et al.* 2023; D'Aloia *et al.* 2016). Interestingly, CFR-T cells exhibit better persistence than traditional CAR-T cells, showing smaller immunological synapses and mitochondrial fusion when recognizing target cells (Cui *et al.* 2023).

Moreover, a protease-based platform for regulating CAR-T-cell activity using an NS3p inhibitor, grazoprevir (GPV), has been designed (SNIP CAR-T). Exposure to GPV prevents CAR cleavage and preserves functional CAR-T cells (ON state). By integrating the cleavage site and protease between the CD8α transmembrane and intracellular signaling domains in a B7H3-targeting CAR, researchers achieved robust GPV-regulated control of CAR cleavage in primary human T cells. SNIP CAR-T cells exhibit greater potency than constitutive CAR-T cells while showing reduced T-cell exhaustion and increased stemness (Labanieh *et al.* 2022).

To address the immunosuppressive effects and enhance control over CAR-T activity in the tumor microenvironment, researchers have developed switchable dual receptor CAR-T cells (sdCAR-T cells). This innovative construct utilizes two antigens, mesothelin and fluorescein isothiocyanate, alongside an FPBM "switch." The FPBM comprises a programmed cell death 1 ligand 1 (PD-L1) blocking peptide conjugated to fluorescein isothiocyanate, effectively regulating sdCAR-T-cell activity. Activation of these cells exclusively occurs in the presence of both FPBM and tumor cells expressing both PD-L1 and mesothelin. Extensive long-term proliferation experiments and pharmacodynamic studies have demonstrated significantly improved antitumor efficacy using this system compared to conventional mesothelin CAR-T cells. The switch-based control offers comparable efficacy with enhanced safety, making it a potential strategy for solid tumor immunotherapy (Yang *et al.* 2020b).

While the mentioned approaches show promise in enhancing control over CAR-T activity, it is important to note that further research is needed to fully understand their potential and optimize their efficacy. Additional studies and clinical trials are underway to evaluate the effectiveness of these strategies in treating solid tumors.

## OPTIMIZING THE SIGNALING DOMAIN OF CAR-T CELLS TO TARGET SOLID TUMORS

### Optimization of the activation domain for improved CAR-T function

In recent years, the limited persistence of CAR-T cells within the immunosuppressive solid tumor microenvironment has been a significant obstacle in the effective use of CAR-T therapy for solid tumors. One promising approach to address this challenge involves modifying the CD3ζ chain immunoreceptor tyrosine-based activation motifs (ITAMs) within CAR-T cells to enhance their activity.

A study evaluated two types of CAR-T constructs. Two conventional second-generation MSLN-CAR T-cell constructs encoding either a CD28 costimulatory (M28z) or 4-1BB costimulatory (MBBz) domain and a novel mesothelin (MSLN)-directed CAR-T-cell construct encoding the CD28 costimulatory domain and CD3ζ chain containing a single ITAM (M1xx) were evaluated using *in vitro* and *in vivo* preclinical models of ovarian cancer (Schoutrop *et al.* 2023). The efficacy of these CAR-T cells was assessed using *in vitro* and *in vivo* preclinical models of ovarian cancer. The results demonstrated that M1xx CAR-T cells exhibited superior antitumor potency and persistence compared to conventional M28z and MBBz CAR-T cells. *In vitro* analysis also revealed that M1xx CAR-T cells had a more favorable phenotype and higher production of key cytokines (IFNγ, TNF, and Granzyme B) associated with antitumor activity. Moreover, M1xx CAR-T cells displayed a self-renewal gene signature, further supporting their enhanced therapeutic potential for ovarian cancer treatment. Another study investigated the effectiveness of the 1XX design, which incorporates a mutated CD3ζ chain containing a single ITAM, in targeting solid tumors (Duan *et al.* 2023). Both CD19- and AXL-specific CARs with the 1XX design were compared to their wild-type counterparts using pancreatic and melanoma mouse models. The 1XX CARs demonstrated better antitumor efficacy in these models, with prolonged persistence of CAR-T cells and a higher percentage of central memory cells. The molecular mechanisms underlying the improved signaling of 1XX CARs were explored using fluorescence resonance energy transfer (FRET)-based biosensors. It was found that decreased ITAM numbers in 1XX CARs led to similar activation of 70-kDa zeta chain-associated protein (ZAP70) but resulted in increased Ca^2+ ^ elevation and faster extracellular signal-regulated kinase (ERK) activation compared to wild-type CARs. In another study (Feucht *et al.* 2019), the modulation of CD28-based CAR-T cells by calibrating their activation potential was explored. By balancing effector and memory programs through the use of a single ITAM, these CAR designs demonstrated enhanced therapeutic profiles in terms of T-cell function and differentiation.

Collectively, these studies emphasize the potential impact of modifying the ITAM domain in CAR-T cells for solid tumor therapy. By fine-tuning CAR-T-cell activation, such modifications could lead to improved antitumor potency, persistence, and functionality, which are crucial for achieving successful clinical outcomes in the treatment of solid tumors.

### Optimization of the costimulation domain to enhance CAR-T-cell activation and persistence

Researchers have explored novel approaches to optimize the design of CAR-T cells for improved efficacy. One such strategy involves incorporating an optimized costimulation domain into the CAR construct. In one study, CAR was engineered by introducing the Toll/interleukin-1 receptor domain of Toll-like receptor 2 (TLR2) to the signaling domain (Lai *et al.* 2018). The introduction of TLR2 signaling resulted in enhanced expansion, persistence, and effector function of T cells expressing CAR. This improvement was observed both *in vitro* and *in vivo* against mesothelin-positive solid tumors. These findings highlight the potential of using TLR2 signaling as a crucial element in enhancing the efficacy of CAR-T cells for solid tumor treatment. However, further clinical trials are needed to establish the safety and efficacy of this approach for solid tumors.

Another vital aspect in optimizing CAR design is the choice of spacer and costimulatory components. A preclinical mouse model was utilized to compare different CAR constructs targeting L1CAM or HER2 antigens (Textor *et al.* 2021). These CAR designs were structurally similar except for the spacer (long or short) or costimulatory (4-1BB or CD28) domains. The study employed a mouse-in-mouse approach, circumventing species-specific factors that affect CAR-T-cell survival, trafficking, and function. This enabled an evaluation of T-cell trafficking, expansion, and tumor control. The results demonstrated that CD28 costimulation exhibited clear superiority in CAR-T trafficking when compared to 4-1BB costimulation. Using L1CAM-specific CAR-T cells, the short spacer-CD28/ζ CAR design showed maximum expansion at the tumor site and initiated tumor regression in mouse xenograft models. These findings were further confirmed by treating patient-derived neuroblastoma xenografts with human L1CAM-targeting CAR-T cells. Additionally, the superiority of CD28 costimulation was also observed in HER2-specific CAR-T-targeting ovarian carcinoma xenografts (Textor *et al.* 2021).

Taken together, these results suggest that incorporating TLR2 and CD28 signaling into CAR design for adoptive T-cell therapy against solid tumors may be advantageous. The optimized costimulation domain contributes to enhanced T-cell trafficking, expansion, and initial tumor regression, paving the way for improved CAR-T treatments. Further research and clinical trials are warranted to fully establish the safety and effectiveness of this approach.

## STRATEGIES FOR ENHANCING CAR-T INFILTRATION INTO SOLID TUMORS

### Approaches to improve CAR-T-cell migration and infiltration

The utilization of CAR-T cells in the treatment of hematological malignancies has displayed noteworthy success due to their ability to effectively infiltrate the circulatory system and bone marrow (Kalos *et al.* 2011). In contrast, their therapeutic efficacy is compromised by the limited infiltration of CAR-T cells within solid tumors. To overcome this challenge, various strategies have been explored with the aim of enhancing CAR-T infiltration into solid tumors.

One such approach involves modulating the expression levels of specific cytokines and chemokines within CAR-T cells to promote their infiltration. Recent studies have shown that overexpression of IL-7 and CCL19 or C-C motif chemokine 21 (CCL21) within CAR-T cells can facilitate immune cell infiltration into tumors and support the persistence of CAR-T cells in the tumor microenvironment (Adachi *et al.* 2018; Luo *et al.* 2020; Pang *et al.* 2021). These findings underscore the significance of cytokine and chemokine signaling in improving CAR-T infiltration and augmenting their antitumor activity for the treatment of solid tumors.

Furthermore, a preliminary study investigating the coexpression of CCR4 and a chimeric antigen receptor targeting CD30 demonstrated enhanced T lymphocyte homing and antitumor activity in a Hodgkin tumor model (Di Stasi *et al.* 2009). These findings suggest that engineered CAR-T cells with specific chemokine receptors might enhance tumor infiltration and consequently improve therapeutic outcomes. Likewise, the engineering of the chemokine receptor CCR2b to coexpress with mesothelin-specific or GD2-specific CAR molecules enhances the trafficking of CAR-T cells in preclinical models (Craddock *et al.* 2010; Moon *et al.* 2011).

Another approach to enhance CAR-T infiltration is the coexpression of CXCR1 or CXCR2 with CARs. CXCR1 and CXCR2 are receptors for interleukin-8 (IL-8), a chemokine primarily produced by cancer cells that is known for its function in attracting polymorphonuclear inflammatory leukocyte infiltrates acting on CXCR1/2 (Alfaro *et al.* 2017). Recent studies have demonstrated that CARs modified with CXCR1/2 significantly enhance the migration and persistence of T cells in various cancers, including glioblastoma, ovarian cancer, pancreatic cancer, and hepatocellular carcinoma (Jin *et al.* 2019; Liu *et al.* 2020; Whilding *et al.* 2019).

In summary, elucidating the mechanisms underlying lymphocyte infiltration mediated by chemokine receptors provides crucial insights into the design of strategies aimed at promoting targeted infiltration of CAR-T cells, thereby improving their antitumor activity in solid tumors.

### Engineering CAR-T cells to degrade the extracellular matrix within the tumor microenvironment

The tumor microenvironment (TME) refers to the complex and dynamic ecosystem that surrounds and supports the growth of tumors. It comprises a variety of different cell types, extracellular matrix components, soluble factors, and physical structures. Among the critical components of the TME are extracellular matrix components, which play a crucial role in shaping tumor progression and influencing therapeutic outcomes. The extracellular matrix is a network of proteins and sugars that provides structural support to cells and also forms a formidable physical barrier within the TME. Dysregulated production and remodeling of the ECM in tumors result in an aberrant composition and architecture, leading to increased tissue stiffness and enhanced interstitial pressure. These changes not only restrict drug penetration but also reduce blood vessel formation, impairing the delivery of CAR-T cells to the tumor microenvironment.

An intriguing discovery has illustrated the role of heparanase in promoting tumor infiltration and augmenting the antitumor activity of CAR-redirected T lymphocytes (Caruana *et al.* 2015). Heparanase degrades heparan sulfate proteoglycans (HSPs), one of the main components of the ECM. This finding suggests that utilizing heparanase as a novel strategy has the potential to enhance the effectiveness of CAR-T therapies by improving infiltration into solid tumors. Another study demonstrated the beneficial effects of human hyaluronidase PH20 on the antitumor activities of mesothelin-specific CAR-T cells against gastric cancer (Zhao *et al.* 2021). By degrading the hyaluronic acid (HA)-rich barriers present in the ECM, PH20 improves the infiltration of CAR-T cells, indicating its potential as an adjunct therapy for enhancing the efficacy of CAR-T treatment in solid tumors.

These studies collectively indicate that engineering CAR-T cells to manipulate enzymatic agents within the tumor microenvironment can significantly enhance CAR-T infiltration and subsequently eradicate tumors. Further research and clinical trials in this field hold tremendous promise for advancing the effectiveness of CAR-T therapy and improving outcomes for patients with various types of solid tumors.

## STRATEGIES FOR OVERCOMING THE IMMUNOSUPPRESSIVE TUMOR MICROENVIRONMENT

**Figure 2:**
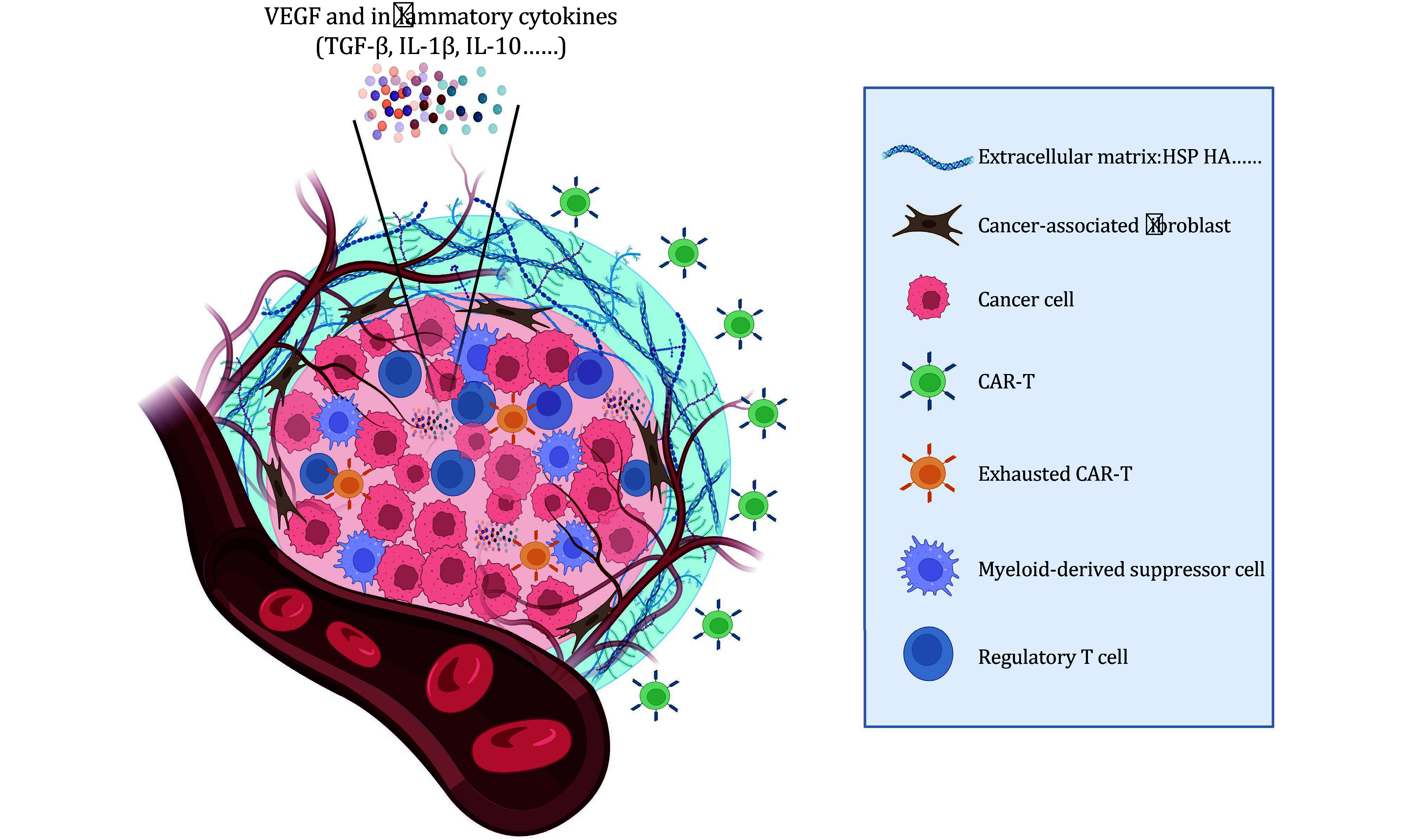
Schematic diagram of the solid tumor microenvironment. TME plays a pivotal role in limiting the efficacy of CAR-T therapy for solid tumors. The TME encompasses a complex network of interactions and conditions within and around the tumor, creating a hostile environment that hinders eradication. Several key factors contribute to this phenomenon. First, the TME consists of immune-suppressive cells, such as regulatory T cells and myeloid-derived suppressor cells. These cells impede the immune response against the tumor, thereby compromising the effectiveness of CAR-T-cell therapy. Additionally, hypoxia, characterized by oxygen deprivation, triggers the expression of VEGF. This leads to the abnormal formation of blood vessels through angiogenesis within the TME. Consequently, there is an inadequate supply of essential nutrients, ultimately compromising immune cell function. Moreover, cancer-associated fibroblasts present in the TME play a dual role. They not only promote tumor growth and invasion but also generate an extracellular matrix that obstructs the infiltration and activity of CAR-T cells. Furthermore, the release of inflammatory cytokines, including TGF-β, IL-1β, and IL-10, creates an inflammatory milieu within the TME that further supports tumor progression. These cumulative challenges posed by the hostile TME underscore the need for innovative approaches to overcome these barriers and enhance treatment outcomes in CAR-T therapy for solid tumors

The TME encompasses a complex network of interactions and conditions within and surrounding a tumor that creates an unfavorable environment for effective immune responses against the solid tumor (Fig. 2). Several factors contribute to immune suppression and evasion, including cancer-associated fibroblasts (CAFs), regulatory T cells (Tregs), and myeloid-derived suppressor cells (MDSCs), which inhibit immune responses and facilitate tumor growth. Additionally, hypoxia, characterized by oxygen deprivation, induces the expression of molecules like vascular endothelial growth factor (VEGF) and abnormal blood vessel formation, leading to nutrient deficiencies and compromised immune cell function. To combat these challenges, researchers have explored various approaches, such as targeting CAFs, modifying CAR-T cells to enhance their functionality, targeting immunosuppressive pathways, and utilizing gene modification techniques. These strategies aim to empower CAR-T therapy in overcoming immune suppression and improving treatment outcomes in combating solid tumors.

### Targeting cancer-associated fibroblasts to disrupt the immunosuppressive niche

CAFs are a type of stromal cell found in the TME of various cancers. CAFs are activated fibroblasts that play a significant role in shaping the tumor microenvironment and promoting tumor growth and progression. They can originate from resident fibroblasts or undergo activation of normal fibroblasts present in the tissue surrounding the tumor. CAFs secrete various signaling molecules, growth factors, and cytokines that support cancer cells’ survival, proliferation, migration, and invasion. They also generate an extracellular matrix that contributes to the formation of a physical barrier around the tumor, making it difficult for therapeutic agents, such as immune cells like CAR-T cells, to penetrate and exert their effects. Furthermore, CAFs interact with other cellular components in the TME, such as immune cells and endothelial cells, through direct cell–cell contact and the secretion of soluble factors. These interactions can further contribute to immune evasion, angiogenesis promotion, and the establishment of an immunosuppressive microenvironment. Due to their critical role in tumorigenesis and tumor progression, targeting CAFs has emerged as a potential therapeutic approach to disrupt the tumor-promoting functions of the TME and enhance the effectiveness of anticancer therapies.

One approach being investigated to clear CAFs is targeting fibroblast activation protein (FAP), which is selectively expressed in immunosuppressive CAFs. FAP has been identified as a candidate universal target antigen due to its reported selective expression in nearly all solid tumors by a subset of immunosuppressive tumor stromal fibroblasts (Bughda *et al.* 2021). A study confirmed this finding, revealing that 18 out of 18 human tumors across various histologies exhibited pronounced stromal element staining for FAP (Roberts *et al.* 2013). Initially, T cells genetically engineered with FAP-reactive chimeric antigen receptors (CARs) demonstrated specific degranulation and production of effector cytokines upon stimulation with FAP or FAP-expressing cell lines (Tran *et al.* 2013). However, further investigations using mice bearing subcutaneous tumors showed limited antitumor effects and associated adverse effects, such as cachexia and lethal bone toxicities, in two mouse strains (Tran *et al.* 2013). This unexpected outcome raised concerns about the use of FAP as a universal target for cell-based immunotherapy. It was discovered that FAP was robustly expressed not only on immunosuppressive tumor stromal fibroblasts but also on multipotent bone marrow stromal cells (BMSCs) in mice and humans (Tran *et al.* 2013). Recognition of these BMSCs by FAP-reactive T cells resulted in lethal bone toxicity and cachexia, further cautioning against the broad use of FAP as a target antigen. Despite these challenges, there is still value in targeting FAP-positive CAFs in the tumor-associated stroma. A study using FAP-specific CAR-T cells demonstrated their ability to recognize and eliminate FAP-positive cells, leading to decreased tumor growth in an A549 lung cancer model (Kakarla *et al.* 2013). Moreover, enhancing antitumor activity was possible by combining FAP-specific CAR-T cells with T cells targeting cancer cells themselves, suggesting the benefit of co-targeting both CAFs and cancer cells in solid tumors. To support these findings, another study utilized a retroviral CAR construct specific for murine FAP, which successfully reduced FAP highly expressed stromal cells and inhibited tumor growth in multiple subcutaneously transplanted tumor types (Wang *et al.* 2014). In the context of malignant pleural mesothelioma (MPM), a devastating disease resulting from exposure to asbestos, FAP expression on reactive tumor-associated fibroblasts makes it an attractive target for adoptive T-cell therapy using CARs (Schuberth *et al.* 2013). FAP-specific CAR-T cells inhibited the growth of FAP-positive human cancer cells in the peritoneal cavity of mice and significantly prolonged the survival of mice. This therapeutic strategy holds promise for treating MPM and other cancers where FAP is highly expressed.

In conclusion, targeting CAFs expressing FAP with CAR-T therapy presents opportunities to overcome the limited efficacy often associated with solid tumor treatment. Cotargeting both CAFs and cancer cells may yield improved outcomes, while considering potential adverse effects, such as lethal bone toxicity and cachexia, is vital for designing safe and effective treatment strategies. Further clinical development of anti-human FAP-CAR is warranted, particularly in the context of malignant diseases where FAP expression is prominent.

### Modifying CAR-T cells to improve their functionality within the hostile tumor microenvironment

In the quest to overcome the challenges posed by hostile tumor microenvironments, researchers have explored various strategies to enhance the efficacy of CAR-T cells designed for solid tumors. One approach involves targeting the interleukin-6 (IL-6) pathway to promote CAR-T-cell proliferation and antitumor activity. By utilizing IL-6 trans-signaling through the GP130/STAT3 pathway, CAR-T cells can be stimulated without systemic IL-6 involvement. CAR-T cells that constitutively express hyper IL-6, a designer cytokine that activates the trans-signaling pathway, exhibited enhanced proliferation and antitumor efficacy *in vitro* and *in vivo*. Constitutively active GP130 expression within CAR-T cells has shown improved antitumor efficacy, offering a potential solution for enhancing CAR-T activity without adverse effects (Jiang *et al.* 2021).

To address the issue of CAR-T exhaustion and improve therapeutic effectiveness, researchers have explored novel CAR-T designs incorporating chimeric switch receptors (CSRs). These receptors combine the extracellular domain of an immune suppressive receptor with an activation signaling domain. By merging inhibitory signals with immune-activating signals, these fusion receptors play a crucial role in enhancing the functionality of T cells within the challenging tumor microenvironment. In a recent study, it was demonstrated that CAR-T cells targeting HER2 and coexpressing CSR specific for PD-L1 exhibited superior fitness and enhanced functions in xenograft models (Ma *et al.* 2022). Further investigations revealed upregulated expression of genes associated with T-cell activation, proliferation, and cytotoxicity in these CAR-T cells. Another study elucidated that coexpression of CSR specific for PD-L1 enhances the antitumor effects of anti-mesothelin CAR-T (CARMz) both *in vitro* and *in vivo*. Interestingly, the coexpression of CSR specific for PD-L1 promotes the differentiation of CARMz T cells into central memory-like T cells, as evidenced by the upregulation of Th1-related genes and downregulation of Th2-associated cytokines mediated through the CD70-CD27 axis (Qin *et al.* 2022). These innovative approaches show promise for overcoming CAR-T exhaustion and maximizing their therapeutic potential.

In the context of B-cell lymphoma, a promising strategy involves coexpressing CD19 CAR with a TGF-β/IL-7 fusion switch receptor. This fusion receptor converts immunosuppressive TGF-β signals into immune-activating IL-7 signals, effectively suppressing B-cell lymphoma recurrence. This approach offers a potential solution for persistent, TGF-β-resistant antitumor treatment (Noh *et al.* 2021).

Recognizing that the immunosuppressive TME hinders solid tumor efficacy and diminishes the anticancer activity of endogenous immune cells, researchers have pursued approaches to enhance CAR-T function within the TME. This includes engineering CAR-T cells to secrete cytokines or express cytokine receptors to modulate the cytokine environment of the TME. A strategy involves secreting antibody-like proteins to target a range of tumor antigens. Collectively known as armored CAR-T therapy, these methods aim to improve the effectiveness of CAR-T cells in the TME (Hawkins *et al.* 2021). Recent studies have shown that CAR-T cells armed with engineered interleukin-2 (IL-2) and interleukin-33 (IL-33) promote tumor control as a single-agent therapy (Brog *et al.* 2022). These armored CAR-T cells synergize to shift leukocyte proportions in the TME, recruiting and activating various innate and adaptive immune cells, including tumor-specific T cells. Depletion of specific cell subsets did not disrupt tumor control, indicating that broad immune activation compensates for the loss of individual cell subsets. This CAR-T platform demonstrates promise in promoting antitumor immunity in multiple solid tumor models and potentially overcoming antigen loss. One of the challenges in utilizing CAR-T for solid tumors is the immunosuppressive TME characterized by high levels of inhibitory factors, such as transforming growth factor (TGF)-β. A phase 1 trial involving castration-resistant prostate cancer-directed CAR-T armed with a dominant-negative TGF-β receptor has shown feasibility and safety. However, future studies should explore multipronged approaches against the TME to further improve outcomes (Narayan *et al.* 2022). Additionally, cytokines, such as interleukin-15 (IL-15), have been investigated to stimulate T-cell proliferation and enhance the activity of CAR-T cells. However, constitutive cytokine signaling can lead to toxicity. Alternatively, interleukin-23 (IL-23), which promotes the proliferation of memory T cells and T helper type 17 cells, has been explored. Engineered expression of the IL-23 subunit p40 in T-cells showed selective proliferative activity and improved antitumor capacity compared to CAR-T cells expressing interleukin-18 (IL-18) or IL-15. This approach demonstrated efficacy in solid tumor mouse models with reduced side effects (Ma *et al.* 2020). In the context of glioblastoma multiforme (GBM), combinational therapies have been explored to overcome the immunosuppressive TME. An orthotopic GBM mouse model revealed that CAR-T cells targeting tumor-specific epidermal growth factor receptor variant III (EGFRvIII) alone were insufficient to control established tumors. However, when combined with a locally delivered dose of interleukin-12 (IL-12), durable antitumor responses were achieved. IL-12 not only boosted the cytotoxicity of CAR-T cells but also reshaped the TME. This promoted proinflammatory CD4^+ ^ T-cell infiltration, reduced regulatory T-cells (Tregs), and activated the myeloid compartment. The benefits of IL-12 were observed with minimal systemic effects, suggesting its potential as an effective adjuvant for CAR-T therapy for GBM (Agliardi *et al.* 2021).

Overall, these innovative approaches, including targeting the IL-6 pathway, utilizing novel CAR designs, developing fusion switch receptors, employing armored CAR-T therapy, and combining CAR-T with cytokines, show promise in overcoming the hostile tumor microenvironment and enhancing the effectiveness of CAR-T therapy for solid tumors.

### Targeting the PD-1/PDL1 pathway to release immune suppression

Tumor microenvironments in solid tumors often contain immune suppressive signals that hinder CAR-T-cell function. Targeting these signals can enhance CAR-T efficacy. For instance, inhibiting immune checkpoint molecules, such as programmed cell death protein-1 (PD-1) or PD-L1, in combination with CAR-T therapy has shown promising results in preclinical and clinical studies (Cherkassky *et al.* 2016; Rafiq *et al.* 2018). Several strategies targeting the PD-1/PDL1 pathway have been explored to overcome this exhaustion and enhance the antitumor activity of CAR-T cells. One approach involves integrating dual immune checkpoint receptors (ICRs) into CAR vectors using lentiviral bicistronic transduction. These vectors include a short hairpin RNA (shRNA) cassette that downregulates PD-1/TIGIT. By downregulating both PD-1 and TIGIT, CAR-T cells demonstrate synergistic antitumor activity. The downregulation of PD-1 enhances short-term effector function, while the downregulation of TIGIT maintains a low differentiation/exhaustion state, resulting in improved therapeutic potential (Lee *et al.* 2022). Another strategy focuses on modifying CAR-T cells to secrete or express PD-1-blocking single-chain variable fragments (scFvs) in the local tumor microenvironment. By combining CAR-T cells with PD-1 scFv, the antitumor activity of CAR-T cells is enhanced. This approach also benefits bystander tumor-specific T cells and improves functionality within the tumor microenvironment. Encouraging results have been observed in clinically relevant PD-L1-positive blood cancer and leukemia mouse models (Rafiq *et al.* 2018). In addition to genetic modifications, the use of PD-1 antibody checkpoint blockade, intracellular PD-1 shRNA blockade, or PD-1 dominant-negative receptor has demonstrated the ability to resist tumor-induced T-cell exhaustion and restore effector function in CAR-T cells (Cherkassky *et al.* 2016). In conclusion, these strategies directly target the PD-1 receptor or its downstream signaling pathways, resulting in improved CAR-T activity.

### Gene modification strategies to enhance the efficacy of CAR-T

Gene modification of CAR-T cells has emerged as a promising approach to enhance their effectiveness in treating solid tumors. Several studies have explored the use of gene editing technologies, specifically CRISPR‒Cas9, to address the limitations of CAR-T therapy and improve their antitumor performance.

The limited clinical efficacy of CAR-T cells in solid tumors has been attributed to PD-1-mediated immunosuppression. In an effort to overcome this limitation, PD-1 and TCR-deficient mesothelin-specific CAR-T (MPTK-CAR-T) cells were generated using CRISPR‒Cas9 technology (Wang *et al.* 2021). Preliminary results from a dose-escalation study involving 15 patients indicated the feasibility and safety of CRISPR-engineered CAR-T cells with PD-1 disruption. Although stable disease was the best overall response, no dose-limiting toxicity or unexpected adverse events were observed. Circulating MPTK-CAR-T peaked within 7‒14 days but became undetectable beyond one month. Interestingly, TCR-positive CAR-T cells were predominantly detected in the effusion or peripheral blood after infusion. Animal models further confirmed the reduced persistence of TCR-deficient CAR-T cells. These findings suggest that the natural TCR plays a crucial role in the persistence of CAR-T cells when treating solid tumors.

Inhibition of immune checkpoints, such as the PD-L1/PD-1 axis mediated by PD-1, contributes to immunosuppression in the tumor microenvironment. Cytokine-inducible SH2-containing protein (CISH), a member of the suppressor of cytokine signaling (SOCS) family, has been found to inhibit JAK-STAT and TCR signaling in T and natural killer (NK) cells. Ablation of CISH in T cells using CRISPR‒Cas9 resulted in increased sensitivity to TCR and cytokine stimulation. Additionally, chimeric antigen receptor T cells lacking CISH exhibited longer survival, higher cytokine secretion, and improved antitumor activity. Activated CISH-deficient T cells displayed decreased expression of PD-1 both *in vitro* and *in vivo*. This decrease in PD-1 expression was attributed to the elevated level of FBXO38, a ubiquitination-regulating protein known to downregulate expression. Inhibition of CISH may therefore present a new strategy for enhancing the therapeutic effect of CAR-T cells (Lv *et al.* 2023).

Another innovative approach involved the development of nonviral, gene-specific targeted CAR-T cells using CRISPR‒Cas9. Through an optimized protocol, researchers successfully inserted an anti-CD19 CAR cassette into the PDCD1 locus, which exhibited a superior ability to eradicate tumor cells in xenograft models. Clinical trials targeting relapsed/refractory aggressive B-cell non-Hodgkin lymphoma showed a high rate of complete remission and durable responses without serious adverse events in patients, even at low infusion doses and with a low percentage of CAR^+ ^ cells. Single-cell analysis revealed that this technique resulted in a high percentage of memory T cells in the infused CAR-T products, indicating the advantages of nonviral, PD1-integrated CAR-T cells (Zhang *et al.* 2022a).

To overcome the challenge of T-cell exhaustion in treating solid tumors, a candidate gene screen was performed using a hypofunction CAR-T model, revealing that depletion of basic leucine zipper ATF-like transcription factor (BATF) improved CAR-T antitumor performance. Loss of BATF in various types of CAR-T and mouse OT-1 cells conferred improved resistance to exhaustion and enhanced tumor eradication efficacy. Mechanistically, BATF was found to regulate the expression of genes associated with exhaustion in human CAR-T cells while also influencing the development of effector and memory T-cells. Knocking out BATF shifted the CAR-T population toward a more central memory subset, underscoring the role of BATF as a key factor limiting CAR-T function. Depletion of BATF enhanced the antitumor activity of CAR-T cells against solid tumors (Zhang *et al.* 2022b).

Suppressive signals from both extrinsic factors and intrinsic inhibitory checkpoints can limit the efficacy of adoptive T-cell therapies for cancer treatment. To address these limitations and enhance T-cell therapeutic function, a study employed multiple genome-wide CRISPR knockout screens and identified RASA2 as a signaling checkpoint in human T cells. RASA2, a RAS GTPase-activating protein (RasGAP), was found to be downregulated upon acute T-cell receptor stimulation and gradually increased with chronic antigen exposure. Ablation of RASA2 resulted in enhanced MAPK signaling and cytolytic activity of chimeric antigen receptor (CAR) T cells in response to target antigen. RASA2-deficient T cells exhibited increased activation, cytokine production, and metabolic activity and demonstrated a marked advantage in persistent cancer cell killing during repeated tumor antigen stimulation. Preclinical models of T-cell receptor and CAR-T therapies confirmed that RASA2-knockout CAR-T cells prolonged survival in mice xenografted with liquid or solid tumors, highlighting RASA2 as a promising target to enhance persistence and effector function in T-cell therapies for cancer treatment (Carnevale *et al.* 2022).

In conclusion, gene modification of CAR-T cells offers a promising avenue for improving their efficacy in the treatment of solid tumors. Various approaches, including CRISPR‒Cas9 technology, have been employed to overcome limitations such as T-cell exhaustion and immune suppression in the tumor microenvironment. Depletion of BATF, ablation of RASA2, disruption of PD-1, and inhibition of CISH have demonstrated enhanced antitumor activity of CAR-T cells in preclinical models. These findings provide valuable insights into the potential of gene modification strategies to enhance CAR-T therapy for solid tumors.

## CLINICAL TRIALS OF CAR-T THERAPY IN SOLID TUMORS

Clinical investigations are currently underway to explore the application of CAR-T-cell therapies for solid tumors. Numerous studies are in the early phase of clinical trials. An interim analysis of an ongoing phase 1 clinical trial focused on CLDN18.2-targeted CAR-T (CT041) revealed promising efficacy and an acceptable safety profile (Qi *et al.* 2022). During this trial, 37 patients were administered one of three doses of CT041: 2.5 × 10^8^, 3.75 × 10^8^ or 5.0 × 10^8^ cells. All patients experienced grade 3 or higher hematologic toxicity, while grade 1 or 2 cytokine release syndrome (CRS) occurred in 94.6% of patients. No significant incidences of grade 3 or higher CRS, neurotoxicities, treatment-related deaths, or dose-limiting toxicities were reported. The overall response rate (ORR) was 48.6%, and the disease control rate (DCR) reached 73.0%. Moreover, the 6-month duration of response rate was 44.8%. Notably, among patients with gastric cancer, the ORR and DCR were 57.1% and 75.0%, respectively. Furthermore, the 6-month overall survival rate for this subgroup was 81.2%.

The cell-surface molecule c-Met was expressed in 50% of breast tumors, prompting the construction of a CAR T-cell specific for c-Met. A subsequent phase 0 clinical trial (NCT01837602) found that in patients with metastatic breast cancer, a single intratumoral injection of 3 × 10^7^ or 3 × 10^8^ mRNA-transfected c-Met-CAR-T cells was well tolerated without any severe side effects. An inflammatory response was observed within tumors following treatment.

In a preclinical study conducted in a mouse model, the expression of IL-7 and CCL19 improved T-cell infiltration and increased the survival of CAR-T cells within mouse tumors (Pang *et al.* 2021). Building upon these findings, a clinical study investigated the use of IL-7- and CCL19-secreting CAR-T cells in the treatment of advanced hepatocellular carcinoma, pancreatic carcinoma, and ovarian carcinoma. This approach showed superior antitumor activity in these patients. Specifically, in a patient with advanced pancreatic carcinoma, treatment with anti-MSLN-7 × 19 CAR-T cells led to the almost complete disappearance of the tumor 240 days after intravenous infusion (Pang *et al.* 2021). These results indicate the potential efficacy of this therapeutic strategy in treating advanced malignancies.

In the context of malignant pleural diseases, a regional delivery approach was adopted for mesothelin-targeted CAR-T-cell therapy followed by the administration of pembrolizumab, a checkpoint inhibitor. This combined treatment demonstrated a good safety profile and exhibited sufficient antitumor efficacy to warrant further investigation (Adusumilli *et al.* 2021). In 27 patients who received intrapleural administration of 0.3 × 10^6^ to 6 × 10^7^ CAR-T cells/kg, the treatment was found to be safe and well tolerated. Moreover, CAR-T cells were detected in the peripheral blood of 39% of patients for more than 100 days, indicating their potential persistence and sustained effects.

Abnormal expression and function of MUC1 can contribute to tumor growth, invasion, metastasis, and evasion of the immune system. Therefore, targeting MUC1 has become an important strategy in therapeutic interventions and diagnostic marker development for certain types of cancers. In the case of a patient with seminal vesicle cancer, an innovative anti-MUC1 CAR-T therapy was administered as part of an interventional treatment (You *et al.* 2016). This therapy involved the use of two distinct anti-MUC1 CAR-T-cell lines. The patient exhibited significant tumor necrosis and positive cytokine responses in their serum, indicating a safe and effective outcome. These initial results are promising.

One challenge encountered in the application of CAR-T cells in solid tumors is the immunosuppressive TME, characterized by high levels of inhibitory factors, including TGF-β. A clinical trial (NCT03089203) aimed at treating castration-resistant prostate cancer enrolled 18 patients who received CAR-T therapy armored with a dominant-negative TGF-β receptor. Among the 13 subjects who received therapy, five developed grade ≥2 cytokine release syndrome (CRS). Unfortunately, one patient experienced grade 4 CRS along with sepsis, resulting in death. Three additional patients achieved a reduction in prostate-specific antigen (PSA) of ≥30%, but CAR-T therapy failure correlated with the upregulation of inhibitory molecules within the TME following cell transfer. Despite these challenges, it was observed that CAR-T therapy armored with a dominant-negative TGF-β receptor was generally safe, with manageable high-grade CRS events (Narayan *et al.* 2022). In contrast, a phase 1 trial (NCT04213469) investigating the impact of PD1-integrated CAR-T cells on B-cell non-Hodgkin lymphoma patients demonstrated high rates of complete remission (87.5%) with no severe adverse events (Zhang *et al.* 2022a). These findings highlight the potential efficacy and safety of this approach in treating this particular type of cancer.

In conclusion, while CAR-T therapy has shown remarkable success in hematological malignancies, its application in solid tumors is a complex and evolving field. Reviewing ongoing clinical trials provides valuable insights into the safety and efficacy of CAR-T therapy in various solid tumor types. Despite these challenges, preliminary findings suggest promising results in certain cancers, urging further research and refinement of the approach for wider clinical use.

## CONCLUSION

CAR-T therapy appears to hold great promise for the treatment of solid tumors. The field of immunotherapy has witnessed remarkable advancements with the development of CAR-T cells. By equipping T cells with CARs, scientists have enhanced their ability to recognize and target specific tumor antigens, resulting in improved tumor eradication. Numerous clinical studies have demonstrated encouraging results (supplementary Table S1 and Table S2), showcasing the potential of CAR-T cells as an effective therapeutic strategy against various types of solid tumors. One significant advantage of CAR-T therapy is its ability to bypass certain immune suppression mechanisms employed by solid tumors. This circumvents many of the limitations faced by traditional treatments, such as radiation or chemotherapy. Additionally, CAR-T therapy offers a personalized approach, as CARs can be engineered to target specific antigens expressed on the surface of cancer cells while sparing healthy tissue. This specificity reduces the risk of off-target effects and improves the overall safety profile of the treatment.

The current focus of research primarily revolves around enhancing effectiveness, but there are areas that require further investigation. These include understanding the complexities of the tumor microenvironment and developing strategies to modulate it in order to enhance CAR-T cells’ function. Overcoming immunosuppression within the tumor microenvironment is also crucial, and exploring combinations with immune checkpoint inhibitors or other immunomodulatory agents holds promise. Additionally, addressing tumor heterogeneity by targeting multiple antigens simultaneously or enhancing CAR-T cells' avidity against diverse tumor antigens may be necessary. Minimizing systemic toxicity, such as cytokine release syndrome and neurotoxicity, while maintaining therapeutic efficacy is a key challenge. Finally, studying resistance and relapse mechanisms and exploring combination therapies will aid in combating resistance and achieving long-term remission. By addressing these unresolved issues and considering future prospects, valuable insights can guide future research and ultimately improve CAR-T immunotherapy outcomes for solid tumors.

Although challenges remain, ongoing research efforts are focused on optimizing CAR-T therapy for solid tumors. The clinical successes observed at the present stage provide a strong foundation for further exploration in the context of solid tumors. As more data emerge from clinical trials and technological advancements continue, the full potential of CAR-T therapy in treating solid tumors is likely to be realized. In conclusion, the bright prospects of CAR-T therapy in the realm of solid tumor treatment make it an exciting area of research and hold promise for improving patient outcomes in the future.

## Conflict of interest

Yuanbin Cui, Mintao Luo, Chuanyuan Gu, Yuxian He, Yao Yao and Peng Li declare that they have no conflict of interest.
